# Surgical Outcomes of Laparoscopic Cystogastrostomy For Pancreatic Pseudocysts: A Retrospective Study

**DOI:** 10.7759/cureus.86476

**Published:** 2025-06-21

**Authors:** Rafique Umer Harvitkar, Seshu Kumar Bylapudi, Ioannis Hannadjas, Jiahui Ren, Ashok Balasubramanian, Yousaf Tanveer, Yasir Alam, Tanwir Jhetam, Giri Babu Gattupalli, Abhijit Joshi

**Affiliations:** 1 General Surgery, Royal Sussex County Hospital, Brighton, GBR; 2 General Surgery, University Hospitals of Leicester NHS Trust, Leicester, GBR; 3 General Surgery, Pilgrim Hospital, Boston, GBR; 4 General Surgery, Belfast Health and Social Care Trust, Belfast, IRL; 5 General Surgery, Metro Heart Institute, Mumbai, IND; 6 General Surgery, Diana Princess of Wales Hospital, Grimbsy, GBR; 7 General Surgery and Endo-Laparoscopy, Dr LH Hiranandani Hospital, Mumbai, IND

**Keywords:** endoscopic drainage, laparoscopic cysto-gastrostomy, pancreatic necrosis, pancreatic pseudocyst, pancreatitis

## Abstract

Introduction

Pancreatic pseudocysts (PP) following pancreatitis pose a management challenge. Radiological and endoscopic methods may result in partial resolution or relapse and are chosen based on pseudocyst size, location, and anatomy. Minimally invasive approaches, particularly laparoscopic cystogastrostomy, are increasingly effective. The laparoscopic transgastric luminal cystogastrostomy technique facilitates complete necrosis debridement and internal drainage with minimal invasiveness.

Materials and methods

We retrospectively reviewed 25 symptomatic, radiologically confirmed patients who underwent laparoscopic cystogastrostomy between 2015 and 2024. Data collected included demographics, cyst characteristics, pancreatitis etiology, BMI, ASA grade, timing, operative details, complications, hospital stay, and return to normal activity. Short- and long-term outcomes were evaluated.

Results

The mean patient age was 55 years (range: 29-80), with 60% male. Gallstones caused PP in 60% of cases. Pseudocysts were located in the pancreatic body (60%) or head/neck (40%). Endoscopy failed in 60% and percutaneous drainage in 40% before surgery. The average BMI was 31 kg/m²; 56% were ASA grade 3. No conversions or perioperative mortalities occurred. The median interval from presentation to surgery was 79 days, with a mean operative time of 135 minutes and blood loss of 150 cc. ICU admission was required in 40% for a mean of 1 ± 0.5 days. The mean hospital stay was 5 ± 1 days.

One patient had a procedure-related complication. At one month, 84% showed complete pseudocyst resolution; the rest resolved within four additional weeks. No recurrences, reoperations, or late complications were observed during a mean nine-month follow-up.

Conclusion

Laparoscopic cystogastrostomy offers a safe, effective, and cosmetically favorable option for PP management, with high resolution and no recurrences in our series.

## Introduction

A pancreatic pseudocyst (PP) is a fluid accumulation rich in pancreatic enzymes that forms in or near the pancreas. It is encased by granulation tissue, which may or may not include fibrous tissue, and does not have an epithelial lining [1].

PPs are a known complication of acute pancreatitis, affecting 3-9% of patients, and chronic pancreatitis, occurs in 12-26% of cases. Treatment is usually required when they become symptomatic, grow larger than 5-6 cm, persist for more than six weeks, or exhibit structural complexity, causing compression on surrounding structures leading to gastric outlet or biliary obstruction, or those that are infected require intervention [1,2]. PP may show no symptoms or manifest through various signs such as abdominal discomfort, a sensation of fullness, nausea, vomiting, or bleeding in the upper gastrointestinal tract [3].

Conventional surgical approaches, such as direct drainage or Roux-en-Y limb drainage, are considered the standard treatment for PP but are associated with significant risks, including a morbidity rate of 22% and a mortality rate of 5%. Minimally invasive methods, including endoscopic drainage and percutaneous drainage, have proven effective in carefully selected patients. Endoscopic drainage provides high early success rates, ranging from 78% to 87%, but carries a recurrence rate of 18-21% and a morbidity rate of 17%. Percutaneous drainage, on the other hand, has a resolution rate of 79%, with a lower recurrence rate of 9%, though it has a complication rate of 19% [4-7].

Currently, advancements in the understanding of the pathophysiology of PP allow for a better selection of patients who are ideal candidates for minimally invasive treatments, aiming to reduce the likelihood of treatment failure and recurrence. Surgical intervention is recommended for patients who do not respond to nonsurgical treatments (endoscopic or percutaneous) or for those whose PP is directly connected to the pancreatic duct [8].

This retrospective study includes 25 patients with PPs who were referred to our tertiary care hospital, some of whom had previously failed ERCP and endoscopic drainage. These patients were treated with laparoscopic cysto-gastrostomy. The advantages of the laparoscopic approach to cysto-gastrostomy and pancreatic debridement, compared to endoscopic methods, include the ability to create a larger anastomosis, achieve better hemostasis of the gastric and cyst walls, and more effectively manage complications like hemorrhage or perforation, while still allowing evaluation and debridement of the inner cyst cavity. This study evaluates the effectiveness of laparoscopic cystogastrostomy in a tertiary care setting, providing insights into long-term outcomes and safety.

## Materials and methods

In this study, we conducted a retrospective review of 25 symptomatic, radiologically confirmed patients who underwent laparoscopic cysto-gastrostomy with internal drainage between October 2015 and October 2024 at Dr LN Hiranandani Hospital, Mumbai. A total of 25 patients (15 males and 10 females) were included in the analysis. The data were retrieved from the hospital’s electronic medical records (EMRs). The diagnosis of PP was based on the patient's medical history, clinical examination, and radiological investigation, mainly computed tomography of the abdomen and pelvis (CTAP). After diagnosis, patients were recommended to complete a pre-anesthesia evaluation along with routine investigations to determine their suitability for surgery and anesthesia. Those patients who met our inclusion criteria underwent surgical procedures.

Only those patients who met the following stringent inclusion criteria were included in the study: (1) symptomatic PP confirmed by contrast-enhanced CTAP; (2) failure of conservative or non-surgical treatments such as endoscopic drainage or percutaneous pseudocyst drainage; (3) medically fit for laparoscopic surgery based on pre-anesthesia evaluation.

Patients were excluded from the study if they met any of the following criteria: (1) coexisting malignancy involving the pancreas or surrounding organs; (2) inability to tolerate general anesthesia due to severe comorbidities or ASA grade ≥ 4; (3) pseudocyst with a complex structure unsuitable for laparoscopic intervention, such as extensive vascular involvement or close proximity to major vessels; (4) pregnancy; (5) previous gastric surgery that altered the anatomy, precluding a laparoscopic cysto-gastrostomy.

Patients were evaluated based on factors including age, sex, cyst location, cause of pancreatitis, body mass index (BMI), ASA grade, time from symptom onset to surgery, operative duration, conversion to open surgery, intra- and postoperative complications, hospital stay, and time taken to resume normal activities. Short- and long-term outcomes were assessed. The patient had a follow-up appointment three months post-surgery with a CTAP, followed by a telephone consultation at six months to assess symptoms.

Relevant clinical data were extracted from patient records and compiled for analysis. Categorical variables were presented as frequencies and percentages, and continuous variables were reported as means and standard deviations or medians and ranges, as appropriate.

Procedure

All surgeries were performed under general anaesthesia with the patient in a supine position, both lower limbs straight and split apart. The surgeon stood between the patient’s legs, while the camera surgeon was positioned on the patient's right side and the scrub nurse on the left.

Pneumoperitoneum was established using the closed technique at the supraumbilical site. A 10 mm supraumbilical optical trocar was carefully inserted, taking precautions to avoid iatrogenic injury. The surgeon’s working trocars (both 5 mm) were then placed: the right-handed trocar in the left hypochondriac region and the left-handed trocar along the right semilunar line, midway between the umbilicus and xiphisternum.

**Figure 1:**
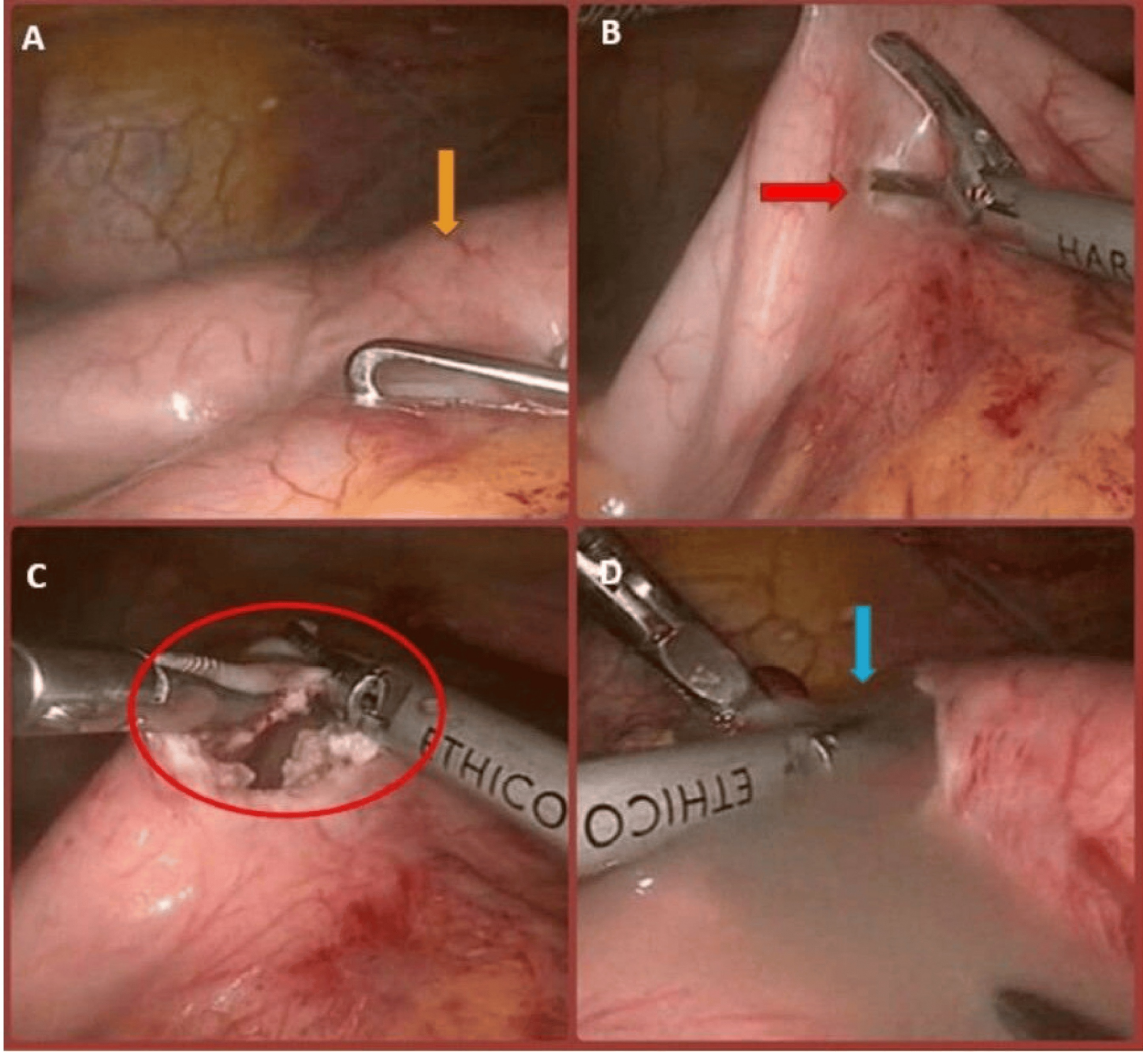
(A) Identification of the pseudocyst bulge on the posterior gastric wall (yellow arrow). (B) Creation of a gastrotomy to access the pseudocyst (red arrow). (C) Evacuation of necrotic debris and fluid contents from the pseudocyst cavity (red circle). (D) Lavage of the pseudocyst cavity following decompression (blue arrow).

**Figure 2:**
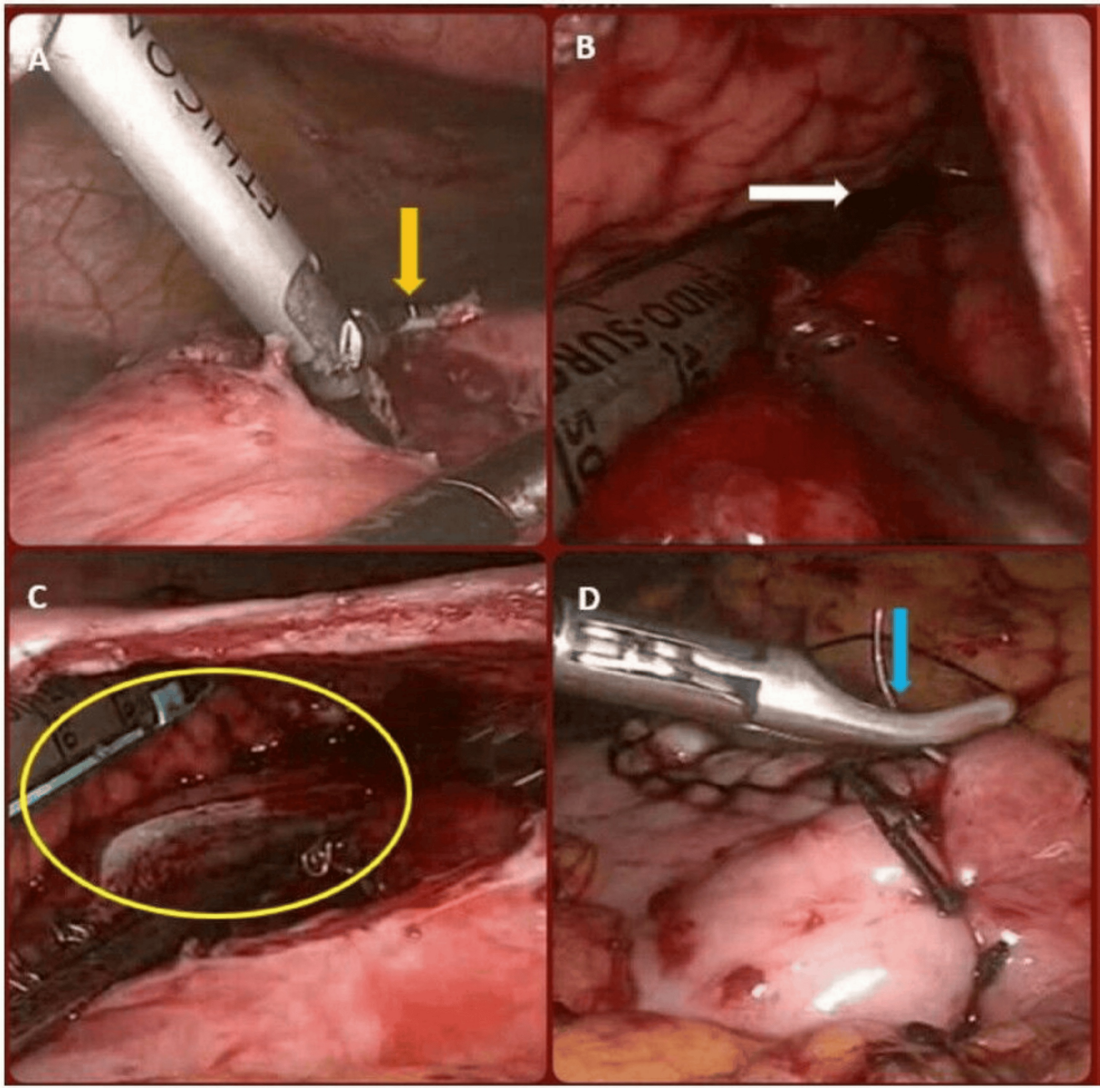
(A) Gastrotomy performed to access the pseudocyst (yellow arrow). (B) Intragastric view demonstrating the pseudocyst cavity (white arrow). (C) Creation of the cystogastrostomy anastomosis using a linear stapler (yellow circle). (D) Closure of the gastric defect with polydioxanone (PDS) sutures (blue arrow).

Upon exploration, a large pseudocyst was observed posteriorly, extrinsically compressing and elevating the stomach (Figure 1A). An anterior gastrotomy was created using a harmonic scalpel (Figure 1B-1C), revealing a bulging mucosal aspect of the posterior gastric wall due to the underlying pseudocyst. After identifying the entry point, the harmonic scalpel was used to incise the posterior gastric wall, allowing immediate drainage of accumulated purulent fluid, which was completely aspirated, followed by lavage (Figures 1D, 2A).

At this stage, the operating and camera surgeons switched positions. The 10 mm supraumbilical optical trocar was replaced with a 12 mm Excel trocar, and the right paramedian 5 mm trocar was upgraded to a 10 mm trocar, which then served as the new optical port. An additional 5 mm trocar was introduced at the epigastric midline.

A 60 mm green cartridge-loaded Endo GIA linear cutter was inserted through the 12 mm trocar, positioning one limb in the pseudocyst cavity and the other in the gastric lumen. The stapler was then fired, creating the cystogastrostomy. After removal of the stapler, the staple line was carefully inspected for haemorrhage (Figure 2B-2C). The pseudocyst cavity was examined to ensure complete evacuation of pus and debris. The anterior gastrotomy was then closed in two layers using 2-0 polydioxanone (PDS) sutures (Figure 2D).

Finally, a peritoneal lavage was performed, pneumoperitoneum was released, and trocar sites were sutured closed.

## Results

The study included 25 patients, with a mean patient age of 55 years (range: 29-80). Males (60%) had a mean age of 47 years, and females (40%) had a mean age of 51 years. The most common etiology was gallstone-induced pancreatitis, 15 (60%), followed by alcohol-related pancreatitis, 5 (20%), hypertriglyceridemia, 3 (12%), and 2 (8%) idiopathic. The average BMI was 31 kg/m², and 14 patients (56%) had an ASA grade of 3. The most common presenting symptom was abdominal pain in 21 (84%) patients, with associated nausea in 13 (52%) cases. Ten (40%) patients also reported weight loss and loss of appetite (Table 1). 

**Table 1:** Patient demographics

Demographic Variable	Value
Total number of patients	25
Mean age	55 years (range: 29-80)
Male patients	15 (60%)
Female patients	10 (40%)
Mean age (male)	47 years
Mean age (female)	51 years
Cause of pancreatitis	
Gallstones	15 (60%)
Alcohol	5 (20%)
Hypertriglyceridemia	3 (12%)
Idiopathic	2 (8%)
Average BMI	31 kg/m^2^
ASA grade 3 or higher	14 (56%)
Most common symptoms	
Abdominal pain	21 (84%)
Nausea	13 (52%)
Weight loss	10 (40%)
Loss of appetite	10 (40%)

The majority of pseudocysts were located in the body or tail of the pancreas (60%), while the remainder were in the head or neck region (40%). They were found to have an average size of 14 ±3.50 cms (range 4.7-22 cms). Concurrent splenic vein thrombosis was observed in three patients (12%).

The median time from symptom onset to surgery was 79 days (range 65-124 days). Prior to surgery, endoscopy failed in 15 patients (60%), while percutaneous drainage was unsuccessful in 10 patients (40%). The mean operative time was 135 minutes, with a median estimated blood loss of 150 cc. No conversions to open surgery were required. Postoperatively, 10 patients (40%) required ICU admission, with an average ICU stay of 1 ± 0.5 days (range: 1-2 days). The average hospital stay was 5 ± 1 days (Table 2). 

**Table 2:** Clinical and operative details ERCP: endoscopic retrograde cholangiopancreatography

Clinical and Operative Detail	Value
Location of pseudocyst	
Body or tail of pancreas	15 (60%)
Head or neck of pancreas	10 (40%)
Average size of pseudocyst	14 ± 3.5 cm (range: 4.7-22 cm)
Splenic vein thrombosis	3 (12%)
Time from symptom onset to surgery	79 days (median)
Failed ERCP	15 (60%)
Failed percutaneous pseudocyst drainage	10 (40%)
Mean operative time	135 minutes
Median estimated blood loss	150 cc
Conversion to open surgery	0 (0%)
Postoperative ICU admission	10 (40%)
Average ICU stay	1 ± 1 day (range: 1-2 days)
Average hospital stay	5 ±1 days

There was no perioperative mortality in this series. Two patients (8%) developed superficial surgical site infections classified as Southampton grades 3a/4a, which required bedside drainage. Additionally, two patients (8%) experienced postoperative pneumonia, while one patient (4%) developed urinary retention. Another patient (4%) had a complicated postoperative course, marked by the development of deep vein thrombosis and pulmonary embolism, both of which were managed appropriately.

All patients experienced resolution of symptoms and fluid collections. At one month postoperatively, 21 patients (84%) showed complete pseudocyst resolution on CT, while four patients (16%) required an additional four weeks for resolution. No cases of pseudocyst recurrence were noted over a mean follow-up period of nine months. Additionally, all patients returned to their baseline activities within an average of four weeks post-discharge (Table 3). 

**Table 3:** Postoperative outcomes

Postoperative Outcome	Value
Postoperative complications	
Superficial surgical site infections	2 (8%)
Postoperative pneumonia	2 (8%)
Urinary retention	1 (4%)
Deep vein thrombosis and pulmonary embolism	1 (4%)
Pseudocyst resolution (1 month post-surgery)	21 (84%) (complete resolution on CT)
Pseudocyst resolution (2 month post-surgery)	4 (16%) (additional 4 weeks for resolution)
Recurrence of pseudocyst	0 (0%)
Mean follow-up duration	9 months
Time to resume normal activities	4 weeks (average)

## Discussion

PPs are encapsulated, fluid-filled collections that arise from pancreatitis, containing pancreatic enzymes, necrotic debris, and occasionally hemorrhagic material. Unlike true cysts, they lack an epithelial lining and are surrounded by a fibrous or granulation tissue wall. These pseudocysts are classified by size, location, and etiology and can be further categorized as acute or chronic based on their association with acute or chronic pancreatitis [8-11]. Their management depends on these classifications, with treatment options tailored to the presence of symptoms, complications, or failure of conservative measures. Pseudocysts in the body or tail of the pancreas are more common, typically associated with acute or chronic pancreatitis due to their proximity to the pancreatic ducts. However, those in the head or neck of the pancreas, though less frequent, present greater challenges due to their proximity to critical structures such as the bile ducts and duodenum, increasing the risk of obstruction and other complications [12,13].

Accurate imaging plays a critical role in the diagnosis and treatment planning of PPs. Contrast-enhanced CT remains the gold standard, while magnetic resonance cholangiopancreatography (MRCP) is particularly useful for evaluating pancreatic ducts and associated abnormalities. Endoscopic ultrasound (EUS) is essential for guiding endoscopic drainage and assessing cyst features, and endoscopic retrograde cholangiopancreatography (ERCP) is utilized for ductal assessment in complex cases. Fine needle aspiration (FNA) may be employed when the diagnosis is uncertain or when malignancy is suspected. Management strategies depend on factors such as cyst size, location, symptoms, and complications, including infection or hemorrhage [14-17]. Small pseudocysts (<4 cm) in asymptomatic patients or those without complications often resolve spontaneously and may not require intervention. Endoscopic drainage is frequently the first-line treatment for symptomatic pseudocysts or those with local complications, achieving high initial success rates (78-87%) but with notable recurrence rates (5-14%). Percutaneous drainage is an option for large or inaccessible cysts or in patients unsuitable for endoscopic or surgical interventions, often serving as a temporary solution. Surgical drainage, particularly laparoscopic cystogastrostomy (LCG), is reserved for cases where less invasive methods fail or when pseudocysts are large, infected, or associated with major complications, such as hemorrhage or bile duct obstruction [13,18].

Both laparoscopic and endoscopic approaches aim to achieve internal drainage of pseudocysts; however, they differ in terms of outcomes and complications. Laparoscopic cystogastrostomy has demonstrated superior long-term results with lower recurrence rates and fewer complications [1,3]. Studies such as those by Bhattacharya and Ammori (2003) and Aljarabah et al. (2007) report recurrence rates of 3-4% with high long-term resolution of symptoms [14,15]. In comparison, endoscopic drainage carries higher complication rates (8.8%), including risks of perforation, bleeding, and infection, as noted by Suggs et al. (2020) [18]. Additionally, endoscopic drainage may be inadequate for larger pseudocysts or those located near major vessels or in the pancreatic tail, as emphasized by Bhattacharya et al. (2003), Sugga et al. (2020), Palanivelu et al. (2007) [14,18,19]. Considering these findings, laparoscopic cystogastrostomy is preferable for patients who have failed endoscopic or percutaneous drainage. It offers lower recurrence rates, fewer complications, and superior long-term outcomes, making it a more effective choice for complex cases. Critics of the laparoscopic transgastric technique often highlight concerns regarding the need for pneumoperitoneum and the potential risks of infection and bowel injury, especially in critically ill patients [18]. However, based on our experience, we did not observe any complications related to pneumoperitoneum tolerance, and no such issues have been reported in the existing literature for this specific technique. Furthermore, there were no infections or bowel injuries in our cohort, and this finding is consistent with ten other case series that similarly reported an absence of these complications. Given these results, it is reasonable to conclude that the risks associated with this technique are likely comparable to those observed in other laparoscopic abdominal procedures [19].

Another criticism of laparoscopic cystogastrostomy is the cost and the requirement for specialized instruments. While this concern may have been valid in earlier years, recent studies, including our own, have shown that standard laparoscopic instruments are sufficient to perform this procedure effectively, without the need for costly, specialized equipment. This development in technique further reinforces the feasibility and cost-effectiveness of laparoscopic cystogastrostomy as a treatment option.

While the results of our study are encouraging, it is important to acknowledge its limitations. The sample size was relatively small, and the follow-up duration was limited. Furthermore, the study was retrospective in nature, and we did not assess post-surgical costs or quality of life. An additional limitation is that the study included only cases that had failed endoscopy, without data on the overall incidence of endoscopic failure. This selection bias may affect the generalisability of our findings, and future studies should aim to address this gap. Despite these limitations, the favourable outcomes of laparoscopic, trans-gastric, endoluminal cysto-gastrostomy in managing PPs and walled-off pancreatic necrosis (WOPN) suggest that it is a promising approach that warrants further investigation. Although a randomized trial may be difficult to conduct for reasons previously discussed, this technique should still be considered a valid and beneficial therapeutic option for treating necrotizing pancreatitis with pseudocyst formation.

## Conclusions

The management of pancreatic pseudocysts requires a tailored, multidisciplinary approach. While small, asymptomatic pseudocysts may resolve spontaneously, symptomatic or complicated cases often necessitate intervention. This study highlights the efficacy of laparoscopic cystogastrostomy, demonstrating lower recurrence and complication rates, particularly in cases where other methods have failed. Treatment decisions should be individualized, considering cyst characteristics and patient factors to achieve optimal outcomes. Advances in imaging and minimally invasive techniques continue to refine management strategies, but further prospective studies with larger cohorts and longer follow-up periods are needed to validate these findings.
